# Hybrid Endo-Epicardial Therapies for Advanced Atrial Fibrillation

**DOI:** 10.3390/jcm13030679

**Published:** 2024-01-24

**Authors:** Christopher X. Wong, Eric F. Buch, Ramin Beygui, Randall J. Lee

**Affiliations:** 1 Centre for Heart Rhythm Disorders, University of Adelaide and Royal Adelaide Hospital, Adelaide 5001, Australia; 2 Cardiac Electrophysiology, University of California San Francisco, San Francisco, CA 94143, USA; 3 Cardiac Arrhythmia Center, University of California Los Angeles, Los Angeles, CA 90095, USA; 4 Cardiothoracic Surgery, University of California San Francisco, San Francisco, CA 94143, USA

**Keywords:** atrial fibrillation, left atrial appendage, catheter ablation, hybrid AF procedure, pulmonary vein isolation

## Abstract

Atrial fibrillation (AF) is a growing health problem that increases morbidity and mortality, and in most patients progresses to more advanced diseases over time. Recent research has examined the underlying mechanisms, risk factors, and progression of AF, leading to updated AF disease classification schemes. Although endocardial catheter ablation is effective for early-stage paroxysmal AF, it consistently achieves suboptimal outcomes in patients with advanced AF. Identification of the factors that lead to the increased risk of treatment failure in advanced AF has spurred the development and adoption of hybrid ablation therapies and collaborative heart care teams that result in higher long-term arrhythmia-free survival. Patients with non-paroxysmal AF, atrial remodeling, comorbidities, or AF otherwise deemed difficult to treat may find hybrid treatment to be the most effective option. Future research of hybrid therapies in advanced AF patient populations, including those with dual diagnoses, may provide further evidence establishing the safety and efficacy of hybrid endo-epicardial ablation as a first line treatment.

## 1. Introduction

Atrial fibrillation (AF) is the most common sustained arrhythmia affecting the general population. It is characterized by a chaotic, rapid, irregular atrial rhythm that results from either electrophysiological and/or structural abnormalities that facilitate this irregular rhythm from cellular connections [1]. The most common origination of the arrhythmia is in the pulmonary veins (PV) or the left atrium [2], and as the disease progresses, fibrotic substrate can further perpetuate the arrhythmia [3]. AF prevalence increases with age, and the lifetime risk of developing AF is approximately one in three, with the highest risk in White individuals, of 30–40% [4,5,6,7]. AF is associated with a five-fold increased risk of stroke and heart failure (HF), a three-fold increased risk of dementia, and approximately 50% greater all-cause mortality [8,9,10,11,12,13,14]. Since treatment of AF has historically been focused on alleviating symptoms, the mortality impact of AF has been under-appreciated, especially in patients over 65 [15]. Although the prevalence of AF appears higher in men than women [15], the risk of mortality may be disproportionately higher in women than in men [16]. Recent studies have examined the impact of gender and cardiac remodeling in AF. Beyer et al. found that in patients with and without AF recurrence, there was a significant difference in baseline characteristics with females presenting with a disproportionately greater rate of recurrence than males [17]. In another study, at the time of ablation, women presented with more advanced structural remodeling despite similar AF duration [18]. Although not modifiable, the higher risk of progression to advanced AF in women should be considered in the treatment of AF and the reduction of AF-related morbidity and mortality.

AF and HF commonly occur together. It can be difficult to determine in a given patient whether AF or HF is the primary problem, since they often cause or exacerbate each other. However, regardless of the etiology, AF is associated with increased risk of cardiovascular hospitalization and mortality, in both the HF with reduced left ventricular ejection fraction (HFrEF) and the HF with preserved left ventricular ejection fraction (HFpEF) populations [19]. In AF patients with HFrEF, HF progresses more rapidly with a rate control strategy and restoration of sinus rhythm results in improved survival and quality of life [19,20].

AF is a progressive disease, and AF begets AF [21]. Previously, AF has been classified based solely on arrhythmia duration, emphasizing treatment options after diagnosis, however a newly proposed classification focuses on the stage of AF with emphasis on prevention, screening, and early treatment to prevent progression to advanced forms of AF. The shifting emphasis to include primary prevention involves identification of modifiable risk factors and implementation of lifestyle changes to reduce the initial onset of AF. After screening and diagnosis of AF, risk factor management remains a key aspect of treatment and prevention of AF-related morbidity. The acronym SOS highlights the three important care processes that must be aligned after the development of AF: Stroke risk assessment and treatment, Optimization of all modifiable risk factors, and Symptom management via rate- and rhythm-control strategies based on individualized care plans [1].

**Figure 1:**
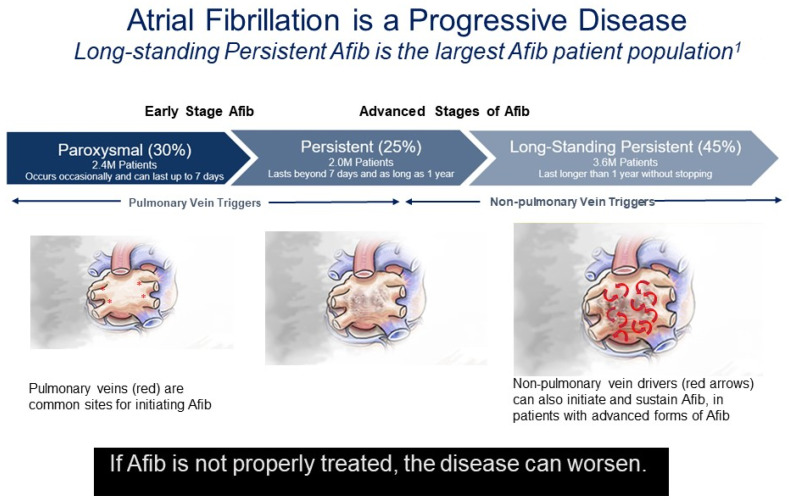
Atrial fibrillation is a progressive disease. Percentages reflect the percentage of diagnosed AF patients in each disease stage of AF Progression from Rahman, F., et al. (2014) [8]. Red asterisks indicate focal drivers. Red arrows indicate random wavelets.

In the absence of effective rhythm control, paroxysmal AF (PAF) usually progresses to more advanced stages of persistent or longstanding persistent AF, although the time course is unpredictable in a given patient (Figure 1). In early-stage PAF, triggering foci in the PV induce most episodes of AF [21]. As AF progresses and left atrial remodeling occurs, other areas such as the left atrial posterior wall (LAPW) and left atrial appendage (LAA) play a more prominent role in initiating and maintaining AF [21]. These areas are commonly the source of foci with rapid ectopic activity, initiating the onset of AF in both structurally normal hearts with paroxysmal AF but also in the reinitiation and maintenance of more advanced forms of AF that may or may not be associated with structural heart disease [2,22]. As far back as 1914, Garrey proposed the critical mass theory, in which a critical left atrial (LA) mass is required for maintenance of AF; as LA size increases there is a greater propensity for sustained AF [23]. This hypothesis has been supported by population-based studies showing a robust association between LA enlargement and increased incidence and prevalence of AF [24,25,26,27,28]. It is unproven if LA enlargement is a cause of AF or consequence of AF, but LA enlargement has been associated with an increased prevalence of AF and poorer outcomes following catheter ablation.

## 2. Treatment Strategies

The treatment of AF has traditionally been focused on the prevention of stroke and the control of AF-associated symptoms as palpitations, fatigue and exercise intolerance. Oral anticoagulation (OAC) therapy is the standard for preventing cardioembolic stroke [29]. In addition to warfarin, which has been commercially available since the 1950s, there are multiple direct oral anticoagulants, which offer similar efficacy and simplified dosing. For patients unable to tolerate long-term OAC, closure of the LAA (the predominant site of AF-related thrombus) with endocardial device implants is an alternative method of stroke prevention [30,31]. There is emerging evidence on minimally invasive epicardial LAA exclusion in this patient population as well, including those not suitable for endocardial LAA occlusion [32,33,34]. 

Two treatment strategies are available for most patients to relieve symptoms related to AF: rate control with atrioventricular (AV) nodal blocking medications, and rhythm control to restore and maintain normal sinus rhythm, usually with antiarrhythmic drugs (AAD) or catheter ablation. All patients with AF, regardless of whether they pursue a rate or rhythm control strategy, should have their stroke risk assessed and appropriately managed. Potential benefits of lifestyle and risk factor management should be considered as part of the prevention of AF and to improve treatment outcomes of patients with AF. Independent cardiovascular risk factors as hypertension, diabetes, coronary artery disease, congestive heart disease and valvular disease have been identified [35]. Male sex, obesity, sleep apnea, left ventricular hypertrophy, and excessive alcohol consumption are additional known associations of AF [36]. Modifiable risk factors as hypertension, obesity and sleep apnea have been shown to affect long-term AF [37]. Therefore, in addition to medical or ablation therapy, risk factor modification is an essential component for AF therapy.

At the earliest stage of paroxysmal AF, avoiding triggers such as alcohol and caffeine, and treating obstructive sleep apnea and other cardiometabolic risk factors can have substantial benefits in reducing AF burden and progression [38]. Historically, the conventional wisdom was that rate control was equivalent to rhythm restoration in terms of cumulative mortality [39,40]. However, this conclusion was largely based on the AF Follow-up Investigation of Rhythm Management (AFFIRM) trial that was reported over 20 years ago, and does not accurately reflect current practice. All patients in the rhythm control arm of the AFFIRM trial were treated with AADs, which have significant toxicity and limited efficacy in maintaining sinus rhythm. Catheter ablation was not available as a treatment option when this study was conducted. Contemporary studies such as the randomized Early Treatment of Atrial Fibrillation for Stroke Prevention Trial (EAST AFNET4) have demonstrated clinical benefits of rhythm control in recently diagnosed AF, even in asymptomatic patients [41]. When added to background therapy of anticoagulation and rate control, rhythm control with AAD or catheter ablation was associated with a lower risk of the primary composite endpoint of cardiovascular death, stroke, and hospitalization due to HF or acute coronary syndrome. Additionally, recent large multicenter randomized trials have demonstrated that rhythm control patients receiving catheter ablation rather than AAD therapy are less likely to progress from paroxysmal to persistent AF [42], and have a significantly lower rate of death or hospitalization for worsening HF [43,44,45]. The benefits of rhythm control are especially pronounced in patients with preexisting HF. As a result of this accumulating evidence on the benefits of rhythm control, there has been a shift to treat AF early with the restoration of sinus rhythm to prevent the consequences of AF progression, rather than just relieving current symptoms [46].

Catheter ablation has been shown to be effective in treating PAF, with success rates at around 70% [47,48,49,50,51], and most patients experiencing a substantial reduction in arrhythmia burden after ablation [52]. However, results of catheter ablation for more advanced forms of persistent and longstanding persistent AF have been disappointing, with success rates of 35–48% at 1 year, and substantially lower at 5 years [53,54,55,56]. As a consequence of these poor results, patients with advanced persistent or longstanding persistent AF are commonly left in AF and treated with only rate control; thus they experience consequences of AF with increased risks of stroke, HF, dementia and mortality [10,15,57,58,59].

Alternative therapies for patients who are not good candidates for rhythm control with AAD or catheter ablation have been relegated to either rate control or AV nodal ablation with pacemaker insertion. Patients treated with rate control commonly remain symptomatic with progression of HF and potential for increased risk of dementia [60,61]. Although AV nodal ablation with pacemaker implantation prevents the rapid rates associated with AF, it does not treat the underlying AF or restore AV synchrony. Patients may still have symptoms associated with AF along with the consequences of increased morbidity and dependency on a pacemaker. Since the procedure cannot be reversed, AV nodal ablation with a pacemaker is generally reserved for symptomatic patients who are refractory to medical management or catheter ablation and in whom rapidly conducted AF is significantly interfering with their quality of life. More recently, hybrid epicardial-endocardial ablation with or without epicardial LAA exclusion has been shown to be superior to endocardial catheter ablation alone for maintenance of sinus rhythm, and may be an appropriate option for more advanced forms of AF [62,63,64]. The remainder of this review will focus on the rationale and potential patient consideration for a hybrid epicardial-endocardial AF ablation approach.

## 3. Factors Leading to Failure of Catheter Ablation in Advanced AF

With advanced forms of AF, electrical and structural LA remodeling often results in LA fibrosis and enlargement. Increased LA surface area increases the potential for the aberrant electrical impulses and reentrant circuits which perpetuate AF. Although PV isolation alone is effective for paroxysmal AF, non-PV structures such as the LAPW and LAA have been shown to play a critical role in the propagation and maintenance of AF in persistent and longstanding persistent AF [65,66,67]. The Cox-maze IV surgical procedure is considered the gold standard for non-PAF and stresses the importance of isolation of the LAPW and LAA exclusion [68].

Non-PAF is commonly associated with changes to the LA substrate [69]. LA enlargement can lead to regional LAPW stress due to pericardial tethering and the LAPW being relatively constrained by the PVs. The regional stress of the LAPW leads to cellular changes as release of atrial natriuretic peptide, calcium overload, increased levels of transforming growth factor β with fibroblast proliferation [69]. Fibrosis occurs, and along with fat tissue, separates myocardial bundles, diminishes cell coupling, and causes slow, anisotropic conduction. Fibrotic infiltration increases the likelihood of areas of heterogeneous conduction and micro and macro-reentrant circuits leading to the genesis and maintenance of AF [70,71]. 

Several recent contemporary prospective randomized studies have investigated the importance of targeting the LAPW [65,72,73]. The ERASE-AF (Low-Voltage Myocardium-Guided Ablation Trial of Persistent Atrial Fibrillation) trial was a multicenter, randomized study of 324 patients randomized in a 1:1 ratio to either PV isolation (PVI) alone or PVI plus substrate modification [65]. Substrate modification consisted of targeting areas of low voltage (atrial voltage < 0.5 mV), which is a surrogate for the presence of arrhythmogenic diseased atrial tissue [74,75]. PVI plus individualized ablation of areas low voltage was significantly improved compared to PVI alone in patients with persistent AF. In contrast, the Stable SR II trial that was similar to the ERASE-AF trial being a multi-center, prospective randomized trial of 300 patients with persistent and longstanding persistent AF randomized in a 1:1 ratio of PVI only versus PVI plus low voltage areas of the LAPW [72]. The study did not reveal any significant difference between the two groups. Similarly, the CAPLA trial, which was a prospective, randomized trial of 328 patients with persistent AF randomized in a 1:1 ration of PVI only versus PVI plus LAPW isolation (roof and floor line), did not demonstrate any significant difference between the two groups [73]. The inconsistent results of endocardial catheter ablation of the LAPW are likely related to many factors such as complex architecture of the LAPW, limitations of current ablation technologies, high recurrence rates regardless of ablation strategy, variations in procedural technique, as well as fear of esophageal injury and atrioesophageal fistula that prevent transmural and durable LAPW lesions.

**Figure 2:**
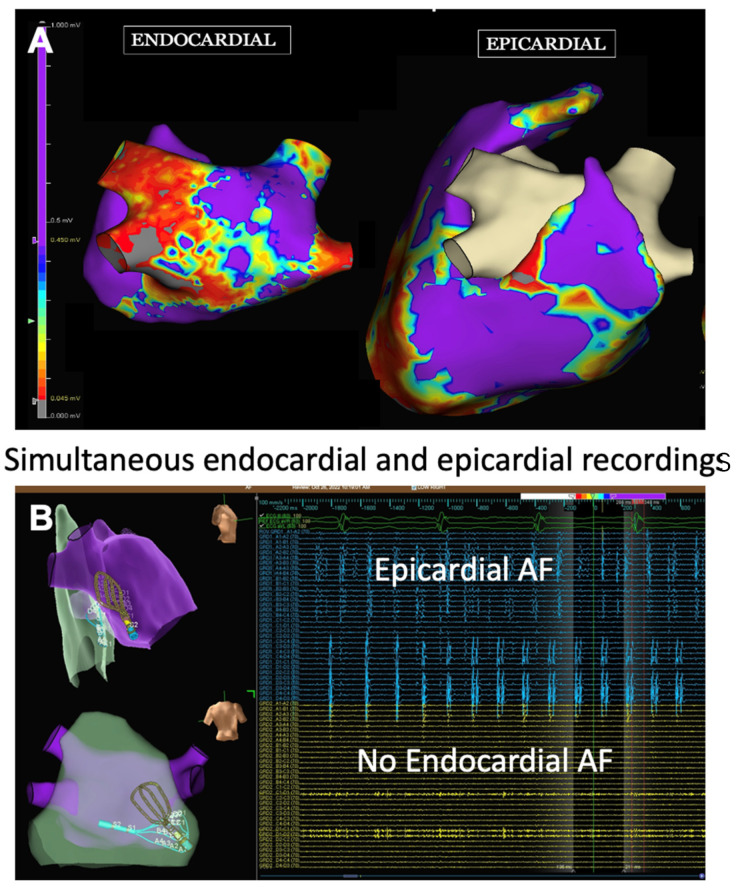
Endocardial and epicardial electroanatomical maps during AF. These images are provided by Dr. Lee and are de-identified images that were obtained for clinical educational purposes as part of standard of care. (**A**) Voltage maps of the endocardium and epicardium of the LA posterior wall. Red (<0.5 mV) indicates scarred areas, while purple (1.5 mV) indicates viable atrial tissue. The endocardial map demonstrates areas of low voltage (red) indicative of scarring while the epicardial voltage map (purple) indicates relatively healthy epicardial tissue. (**B**) Simultaneous endocardial mapping and epicardial mapping with a multi-electrode grid catheter (Abbott, Inc., Minneapolis, MN, USA). The upper left figure (right lateral view) and lower left figure (posterior view of the LA posterior wall) of the endo-epi maps demonstrating that the endocardial grid and epicardial grid are overlayed in the same area of the LA posterior wall. There are no endocardial electrograms (yellow) in the lower right figure which corresponds to the scarred area of the endocardial voltage map seen in figure (**A**). The epicardium (blue) figure in the upper right demonstrates a high amount of epicardial electrograms demonstrating that the epicardial layer of the LA posterior wall is in AF while there is no AF in the endocardial layer.

The LAPW is a complex structure consisting of variable fiber orientation and wall thickness ranging from 1.5 mm to 6.5 mm, and is more similar embryologically and electrophysiologically to PV tissue than atrial tissue [76,77]. The bundles of LAPW myocytes, fibrosis, LA stretching, and adipose infiltration associated with LA remodeling during advanced AF lead to complex LAPW physiology in which there are epicardial to endocardial bundle connections and a physiological separation between the endocardial and epicardial layer that enables the two layers to act independently [78]. This epicardial-endocardial LAPW electrical dissociation can lead to electrically isolated endocardium, while the adjacent LAPW epicardium can still maintain AF (Figure 2) [79,80]. The commonly used energy sources for catheter ablation do not consistently create transmural lesions in the thickest regions of the LAPW or those near the esophagus, so endocardial catheter ablation may result in endocardial LAPW electrical isolation without isolating LAPW epicardial tissue that maintains AF.

The LAA is another potential substrate for AF. The LAA is a highly trabeculated structure and is dominated by extensive pectinate muscles [81]. The heterogeneous fiber orientation creates anisotropy, influences propagation and favors the formation of localized reentry [82]. The LAA has been shown to be the site of complex fractionated electrograms and the maximal in the majority of patients with persistent AF [83,84]_._ The elimination of dominant frequency of >11% has been shown to be associated with the maintenance of sinus rhythm after the ablation of persistent AF [85].

Focal triggers arising from the LAA and LAA have been associated with initiating AF [67,86,87]. In patients with persistent and longstanding persistent AF, triggers arising from the LAA have been observed in 23% of patients with persistent AF and 58% of patients with longstanding persistent AF [66]. Electrical isolation of the LAA increases the efficacy of catheter ablation for persistent and longstanding persistent AF [66]. However, LAA electrical isolation by catheter ablation is difficult to achieve, with high recurrence rates and potential complications such as LAA perforation or phrenic nerve injury [88]. When electrical isolation of the LAA is achieved, the resulting mechanical standstill of the LAA may be associated with higher incidence of LAA thrombus formation and embolic stroke risk despite OAC [89,90]. 

An alternative approach to addressing the arrhythmogenic and embolic potential of this structure is epicardial LAA exclusion [91,92,93]. Complete epicardial LAA exclusion eliminates not only electrical activity within the LAA but also blood supply, eventually resulting in ischemic necrosis, atrophy and resorption of the LAA, which is analogous to LAA surgical amputation. The potential benefits of epicardial LAA exclusion include elimination of the most important nidus for thrombus formation, LAA electrical isolation and decreased LA volume [93,94,95,96,97].

## 4. Hybrid AF Therapies

Although surgical treatment of AF is well established as a concomitant procedure during heart surgery, more recently a less invasive hybrid epicardial-endocardial approach for the treatment of persistent and longstanding persistent AF has been developed. Hybrid AF ablation requires a team approach in which a cardiac surgeon and cardiac electrophysiologist work together to perform a two-stage procedure: first epicardial ablation under direct visualization via a minimally invasive approach, which also permits LAA exclusion, followed by second-stage percutaneous endocardial catheter ablation, either on the same day or several weeks later. The epicardial ablation approach has the advantages of being able to access epicardial structures with direct visualization and safely ablate the LAPW epicardium by directing energy towards the heart and away from the esophagus, thus minimizing the risk of esophageal injury. Subsequent endocardial catheter ablation utilizes electroanatomical mapping to complete PVI, identify lesion gaps, and ablate additional areas such as the LA roof, which is difficult to fully access from the epicardial surface due to pericardial reflections. Taken together, the goal of the combined hybrid epicardial-endocardial ablation approach is to create more consistent transmural lesions and durable LAPW isolation by ablating both atrial surfaces. Additionally, a potential benefit of epicardial LAA exclusion is the elimination of a potential source of thrombus formation and arrythmias arising from the body of the LAA [67,94,95,96]. Furthermore, in patients with LA enlargement, reduction of the LA electrical mass could provide a benefit in rhythm control [98,99].

Minimally-invasive epicardial surgical ablation alone has shown inferior results to hybrid procedures with no significant benefit compared to catheter ablation alone [100,101,102,103]. Atrial arrhythmia recurrence rates of 40% have been noted with only epicardial ablation due to incomplete transmural lesions [102]. Incomplete transmural lesions can lead to slowed conduction without obtaining a conduction block, thus creating the substrate for focal micro reentrant atrial tachycardias or atypical atrial flutters. The incomplete lesion gaps produced by epicardial only ablation can be remedied by endocardial mapping to identify the gaps and endocardial ablation to complete the lesion.

**Figure 3:**
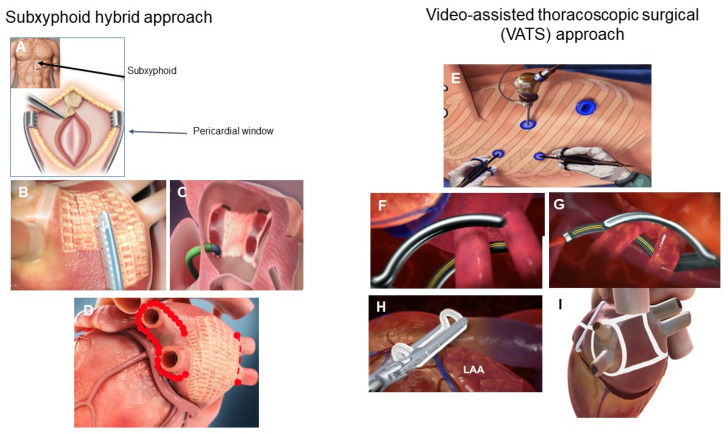
Hybrid AF surgical approaches. The predominant hybrid AF surgical approaches utilize the subxiphoid approach (**A**) or the video assisted thoracoscopic surgical approach (VATS) (**E**). In the subxiphoid approach, a small 3–4 cm incision is made below the xyphoid process (**A**) to allow for the creation of a pericardial window. Left atrial posterior wall ablation is performed with the EPi-Sense ablation catheter (AtriCure, Inc., Mason, OH, USA) (**B**). During stage 2 of the procedure, endocardial mapping and ablation is performed (**C**) to complete any gaps and to complete the pulmonary vein isolation. A typical subxiphoid hybrid ablation approach consists of epicardial left atrial posterior wall isolation (waffle pattern, (**D**)) and endocardial catheter ablation to complete the pulmonary vein isolation (red dots, (**D**)). The VATS hybrid AF approach generally consists of sequential right and left pulmonary vein isolation with radiofrequency ablation clamps (**F**,**G**), placing of an AtriClip for LAA exclusion (AtriCure, Inc., Mason, OH, USA) (**H**), excision of the ligament of Marshall and creation of a roof and floor line to create a “box” left atrial posterior wall isolation. The minimal lesion set is pulmonary vein isolation, isolation of the left atrial posterior wall, excision of the ligament of Marshall and LAA exclusion (**I**).

There are two predominant minimally invasive surgical approaches used for epicardial ablation: via a subxiphoid incision or a video-assisted thoracoscopic surgical (VATS) approach (Figure 3) [104,105]. Recent prospective randomized controlled trials (RCT) of both approaches have reported statistically superior results for hybrid epicardial-endocardial ablation versus endocardial catheter ablation, with no significant difference in adverse event rates (Table 1) [62,63,64]. In general, cryotherapy and radiofrequency catheter ablation have been used predominantly in PAF and early persistent AF. Catheter ablation for more advanced forms of persistent and longstanding persistent AF have been disappointing, with success rates of 35–48% at 1 year and substantially lower at 5 years [53,54,55,56]. Despite various ablation strategies and lesion sets, there has been no reduction in the rate of recurrent atrial arrhythmias as compared to PVI [106]. Therefore, endocardial catheter ablation has been ineffective in treating advanced forms of persistent and longstanding persistent AF.

Three randomized clinical trials have been published comparing the effectiveness and safety of hybrid ablation to endocardial catheter ablation alone for persistent and longstanding persistent AF [62,63,64]. The CONVERGE trial was a prospective, multicenter, 2:1 randomized clinical trial of 153 patients at 21 sites comparing same-day hybrid convergent ablation (without LAA exclusion) and catheter ablation in patients with persistent and longstanding persistent AF undergoing their first ablation [64]. The final lesion set for the hybrid therapy in the CONVERGE trial consisted of PVI, LAPW isolation, and a cavotricuspid isthmus ablation. The control endocardial catheter ablation lesion set consisted of PVI, a roof line, and a cavotricuspid isthmus ablation. Through 12 months, freedom from AF/AFL/AT off new AADs or increased doses of previously failed AADs was 67.7% (67/99) in the Hybrid arm and 50.0% (25/50) in the Catheter arm (*p* = 0.036), representing an absolute benefit of 17.7% with hybrid ablation and meeting the primary effectiveness endpoint. The safety endpoint was also met, with 2.9% through 7 days and a 7.8% (8/102) rate of major adverse events within 30 days after hybrid ablation; half of these events were delayed inflammatory pericardial effusions including one with major bleeding. Forty-two percent (n = 65) of the patients in CONVERGE had longstanding persistent AF (continuous AF greater than 1 year in duration), and a post-hoc analysis of this subgroup found significantly greater freedom from AF/AFL/AT of new AADs or increased dose of previously failed AADs through 12 months in the hybrid arm compared to the catheter arm [107]. At 18 months, 68% of patients with longstanding persistent AF remained free of recurrence when treated by the hybrid ablation via a subxiphoid approach, compared to 30% of patients treated with endocardial ablation. By making more consistent and durable transmural lesions of the LAPW, the hybrid approach results in reduced surface area able to sustain micro- and macro-reentrant circuits that perpetuate AF, especially when combined with LAA exclusion. In addition to increased freedom from atrial arrhythmias with a hybrid epicardial/endocardial ablation approach, patients have demonstrated significant quality of life improvement with the hybrid approach compared to catheter ablation alone [108]. 

The CEASE-AF trial is the largest prospective randomized controlled trial comparing hybrid ablation treatment approach consisting of combined thoracoscopic epicardial ablation, LAA exclusion and endocardial catheter ablation to a treatment approach consisting exclusively of endocardial catheter ablation, including clinical indicated repeat catheter ablation within 6 months of the index procedure [62]. The CEASE-AF trial consisted of 170 patients with a history of symptomatic persistent AF and a LA diameter (LAD) > 4.0 cm or symptomatic LSPAF; and had failed at least one class I or III AAD who were randomized in a 2:1 fashion to either VATS hybrid ablation or catheter ablation. The minimal lesion set of the hybrid population consisted of PVI, LAPW isolation by means of a “box” lesion (i.e., superior and inferior connecting lines between right and left PVI) and LAA exclusion with an AtriClip or stapler. The control catheter ablation group had a minimal lesion set of PVI with additional ablation strategies per the physician’s discretion/center’s standard, which resulted in more than 40% of the control group also undergoing LAPW isolation. At 12 months, freedom from atrial arrhythmias was 71.6% in the hybrid group and 39.2% in the control group resulting in an absolute difference of 32.4% and a relative reduction of 82.7%. In a post-hoc analysis of the longstanding AF population, freedom from atrial arrhythmias was 66.7% in the hybrid group and 25% in the catheter ablation group for an absolute difference of 41.7% with a relative reduction of 166.8%. There was no statistical difference in major complications nor total adverse events between the groups. 

The HARTCAP study was a single center prospective randomized trial of 41 ablation-naive patients with long-standing persistent AF randomized in a 1:1 fashion to hybrid therapy or catheter ablation [63]. The hybrid lesion set consisted of pulmonary vein isolation, LA posterior wall box lesion and LAA exclusion with either the AtriClip or LARIAT closure device (AtriCure, Inc., Mason, OH, USA). The lesion set for the catheter ablation arm was pulmonary vein isolation and LA posterior wall box lesion. At 12 months, freedom from atrial arrhythmias in the hybrid AF was 89% versus 41% in the catheter ablation arm. Of note, the HARTCAP population had severely enlarged LA with a mean LAVI of 54 mL/m^2^ for the hybrid group and a LAVI of 49 mL/m^2^ for the catheter ablation group. The results of the CONVERGE, CEASE AF and HARTCAP prospective randomized studies consistently demonstrate that hybrid epicardial-endocardial ablation significantly reduces recurrence of atrial arrhythmias compared to catheter ablation, particularly in longstanding AF [62,63,64,107].

A benefit of a VATS hybrid procedure for epicardial ablation or exclusion of the LAA with an AtriClip is that it provides access to the ligament of Marshall (LOM). The LOM is an epicardial structure that traverses between the left upper PV and the LAA. It is a known source of triggers for AF and due to its extensions into the lateral wall and coronary sinus, the LOM has been associated with both focal atrial tachycardias and atypical atrial flutters [109,110]. The prospective randomized VENUS trial demonstrated a significant increased likelihood of remaining free of AF or atrial tachycardia when combining ethanol injection into the vein of Marshall with catheter ablation as compared to catheter ablation alone [111]. Since the LOM is readily accessible during a VATS procedure, excision or ablation of the LOM can be easily performed.

## 5. Collaborative Heart Team Approach to Hybrid Epicardial-Endocardial Ablation

A collaborative heart team approach to hybrid epicardial-endocardial ablation was first published in 2010 by Andy Kiser and colleagues, who proposed to converge epicardial and endocardial ablation patterns into a single AF treatment method performed by two specialties—cardiac surgery and electrophysiology [112]. As such, the hybrid convergent procedure leveraged the anatomical approach taken by the cardiac surgeon and the physiological approach taken by the electrophysiologist to facilitate better procedural outcomes and communication amongst the collaborating physicians to treat AF. Individually, neither approach can achieve complete transmurality due to thickness of the tissue, however taken together, the goal of the combined approach is to create overlapping and contiguous lesions to create a transmural barrier to abnormal conduction. The role of the collaborative patient-centered, multi-disciplinary heart team is vital in determining the appropriate patients for consideration of hybrid therapies as well as ensuring an optimal risk–benefit assessment and maximizing treatment outcomes for historically difficult to treat patient populations [113,114].

## 6. Potential Candidates for Hybrid AF Therapies

**Figure 4:**
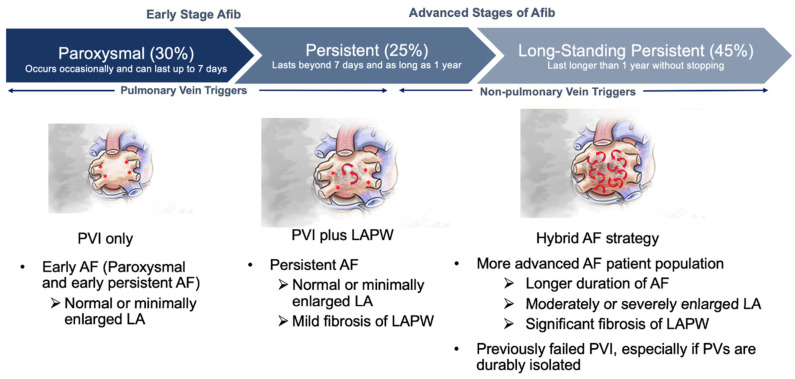
Potential patient for consideration for hybrid AF strategy. Red asterisks indicate focal triggers. Red arrows indicate random wavelets.

Patients with enlarged atria and advanced non-paroxysmal AF have high recurrence rates following catheter ablation [115,116]. The Efficacy of Delayed Enhancement-MRI-Guided Fibrosis Ablation vs. Conventional Catheter Ablation of Atrial Fibrillation (DECAAF II) sub study demonstrated that LA volume (LAV) was the strongest predictor of atrial arrhythmia recurrence after PVI in persistent AF patients [117]. Patients with a LAV less than 114 cc had 73% one-year freedom from recurrence of atrial arrhythmias, whereas patients with LAV greater than 114 cc had 53% freedom from recurrence of atrial arrhythmias, which worsened as LAV increased [117]. These findings were corroborated by the prospective randomized Left Atrial Appendage Ligation With the LARIAT™ Suture Delivery System as Adjunctive Therapy to Pulmonary Vein Isolation for Persistent or Longstanding Persistent Atrial Fibrillation (aMAZE) trial, in which the prespecified variable of LA size was associated with recurrence of AF following PVI. LA volume of 130 cc was associated with PVI efficacy of 53% with decreasing efficacy with increasing LAV; while the LAA ligation plus PVI group had efficacy rates of 65% which remained relatively constant despite increasing LAV [97]. Additionally, patients with persistent or longstanding persistent AF have a high rate of AF recurrence in the range of 60–65% following endocardial catheter ablation [118,119]. The low success rates of catheter ablation alone in patients with advanced AF and LA enlargement, coupled with the randomized hybrid AF trials, suggest that these patients should be considered for hybrid therapy (Figure 4). Another group of patients that do not respond well to catheter ablation alone are patients that have previously undergone catheter ablation in which the pulmonary veins have already been isolated. The PARTY PVI study investigated patients with durable PVI who underwent various catheter ablation strategies to eliminate the recurrence of AF [120]. Regardless of the catheter ablation strategy, there was no strategy that was superior in improving freedom from atrial arrhythmias. LA dilatation was a significant predictor of recurrence of atrial arrhythmias. A hybrid AF ablation strategy with epicardial LAA exclusion may be a reasonable option for these repeat catheter ablation patients. 

Patients with longstanding persistent AF (AF > 1 year) could be considered for hybrid therapy as a first line therapy. The post hoc analysis of the prospective randomized CONVERGE trial of the longstanding persistent AF population in which the mean AF duration was 6.0 years demonstrated an absolute difference of 28.8% between the subxiphoid hybrid approach compared to catheter ablation (65.8% versus 37.0%, respectively) which is a 78% improvement in freedom from atrial arrhythmias [107]. The totality of evidence and the risk–benefit profile of the Convergent subxiphoid hybrid procedure has led to the only FDA-approved ablation device for longstanding persistent AF to be used in a hybrid epicardial-endocardial approach. Another consideration for the use of hybrid therapy for first line therapy would be in patients with enlarged LA and late persistent to longstanding persistent AF. As seen with in the CONVERGE trial, both the CEASE-AF and HARTCAP prospective randomized trials demonstrated that patients with longstanding persistent AF had a significant reduction in atrial arrhythmias at 12 months following a VATS hybrid procedure versus catheter ablation [62,63]. Additionally, the patients in the HARTCAP trial had a mean transthoracic echocardiogram LAVI diameter of 54 mL/m^2^ [63]. The VATS hybrid arm had over an 85% efficacy rate of freedom from arrhythmias while catheter ablation 12-month efficacy was 41%. Combined with the data from the prospective randomized DECAAF and aMAZE trials where freedom from atrial arrhythmias were below 50% in patients with moderate to severely enlarged LA (magnetic resonance imaging or computerized tomography LA volumes greater than 130 cc) suggests that this patient population would benefit from hybrid therapy as a first line therapy [97,117].

Although the recent prospective randomized studies on hybrid therapy suggest that hybrid therapy could be used as first line therapy for longstanding persistent AF or in advanced AF with moderate to severe LA enlarged, some patients and cardiac electrophysiologist prefer to have at least one attempt at endocardial ablation to avoid the potential morbidity and longer recovery time associated with the hybrid approach. Since hybrid procedures consist of epicardial ablation, commonly with LAA exclusion, and a separate endocardial ablation, the order of the hybrid procedure could be reversed. A benefit of reversing the order is that there will be a low percentage (below 40%) of patients that may have an adequate response to endocardial ablation and not undergo the minimally invasive surgical part of the hybrid procedure. The limitation of this strategy should the patient not respond to endocardial catheter ablation alone is that after the epicardial ablation, there may need a “touch up” endocardial ablation to complete gaps in the epicardial ablation lesions.

A factor to be considered before recommending a hybrid AF procedure is whether a patient can tolerate the procedure. Hybrid procedures, especially VATS approach or involving an AtriClip for LAA exclusion require single lung ventilation which preclude patients with severe pulmonary disease. Recent randomized trials indicate that mortality and severe adverse events are similar between hybrid patients versus catheter ablation only, however there are potential adverse events that may be more prevalent with a hybrid procedure. Since hybrid procedures enter the pericardial space and the LAA is commonly excluded, pericarditis and potentially delayed inflammatory pericardial effusions or pleural effusions can occur. Pericarditis and delayed pericardial effusions have been mitigated with the prophylactic use of colchicine and nonsteroidal anti-inflammatory medications [105]. Post-operative transthoracic echocardiography has been recommended prior to discharge and 1–3 weeks post-procedure to assess for late pericardial effusions [105]. Similarly, post-operative pain associated with VATS port placement should be addressed with adequate pain medications [105]. Post-operative pain management and assuring adequate lung reinflation generally leads to a 2–3 day hospitalization as compared to same day or overnight stay for catheter ablation.

Hybrid epicardial-endocardial AF ablation can be performed at the same setting. However, the majority of hybrid procedures are staged with the epicardial ablation and LAA exclusion done as the first stage with endocardial ablation being performed at a later time period. Thus, patients do undergo two separate procedures. Hybrid procedures are commonly performed in the operating room or hybrid room under general anesthesia, so both the coordination of the procedure as well as comorbidities have to be taken into account. Since VATS surgical approaches require specific training and expertise with thoracoscopic surgery and can be technically challenging, not all centers are equipped. The use of a subxiphoid hybrid approach has simplified the technical aspects of the procedure, but still requires coordination between the cardiac electrophysiologist and cardiovascular surgeon and generally is performed as two procedures [121].

Pulsed field ablation (PFA) has emerged as an alternative energy source to radiofrequency or cryotherapy for catheter ablation. Pulse field ablation has the potential benefit of myocardial tissue being preferentially ablated while sparing ablation of adjacent tissues. Initial clinical results demonstrate safety, especially the lack of permanent phrenic nerve injury or atrioesophageal fistulas, while having similar long-term efficacy as compared to radiofrequency ablation and cryotherapy [47,122,123]. The speed and perceived safety of PFA makes it an attractive form of catheter ablation to extend beyond the pulmonary veins to include the LA posterior wall [124]. PFA may also be coupled with hybrid therapies in the future. Due to the safety and speed of PFA, one could consider the same setting to perform hybrid epicardial-endocardial ablation, thus eliminating the need for a staged procedure.

## 7. Conclusions

As the most common sustained arrythmia, AF is a global public health problem with considerable morbidity and mortality risks. Certain patient populations may be at a higher risk of developing AF and/or progressing to advanced stages of disease. Adaptations to the AF classification scheme have shifted the focus to include primary prevention and risk mitigation to remain a part of treatment strategies. Although most patients with AF benefit from rhythm control, this has been difficult to achieve with AADs and endocardial catheter ablation, especially for those patients with clinically advanced AF. Hybrid ablation achieves higher success rates in such patients, likely due to elimination of triggers and substrate in the LAPW and LAA, as well as more durable and transmural lesions. This approach also offers mechanical exclusion of the LAA, eliminating an important source of thrombus formation and potentially thromboembolic events. An individualized collaborative care team approach is essential to the proper treatment of AF. While some patients may opt to attempt endocardial only ablation before potentially moving to hybrid therapy treatment options, hybrid therapy as a first line treatment should be a consideration for patients with advanced AF.

## 8. Future Directions

Future research should explore the use of hybrid therapies in patients with advanced AF and other comorbidities. A meta-analysis of the existing recent research would provide strong evidence and would be a welcome addition to the published literature.

## Figures and Tables

**Table 1:** Randomized trials comparing hybrid approach to catheter ablation in persistent and longstanding persistent AF.

	Trial		
	CEASE * LSPersAF n = 30 PersAF n = 124	HARTCAP ** LSPersAF = 41	CONVERGE * LSPersAF n = 65 PersAF = 88
Inclusion Criteria	Age: 18–75 years Symptomatic PersAF/LSPAF LAD > 4.0 cm	Age: ≥18 years Symptomatic PersAF/LSPAF LAD ≤ 60 mm	Age: 18–80 years Symptomatic PersAF LAD ≤ 6 cm
Procedural Access Method	Bilateral thoracoscopy	Unilateral or bilateral thoracoscopy	Subxiphoid
Hybrid arm	71.6% (68/95)	89.0% (17/19)	67.7% (67/99)
Catheter ablation arm	39.2% (20/51)	41.0% (9/22)	50.0% (25/50)

PersAF: persistent AF; LSPersAF: longstanding persistent AF. * Primary effectiveness defined as freedom from AF/AFL/AT > 30 s absent class I/III anti-arrhythmic drugs (AADs), except those previously failed with no increased dose; CONVERGE did not include epicardial LAA exclusion; ** Primary effectiveness defined as freedom from any atrial tachyarrhythmia > 5 min off AADs.
